# The Receptor for Advanced Glycation End-Products (RAGE) Is Only Present in Mammals, and Belongs to a Family of Cell Adhesion Molecules (CAMs)

**DOI:** 10.1371/journal.pone.0086903

**Published:** 2014-01-27

**Authors:** Luca Sessa, Elena Gatti, Filippo Zeni, Antonella Antonelli, Alessandro Catucci, Michael Koch, Giulio Pompilio, Günter Fritz, Angela Raucci, Marco E. Bianchi

**Affiliations:** 1 Chromatin Dynamics Unit, San Raffaele University and Research Institute, Milano, Italy; 2 Laboratory of Vascular Biology and Regenerative Medicine, Centro Cardiologico Monzino, Milano, Italy; 3 Institute of Neuropathology, University of Freiburg, Freiburg, Germany; University of Miami, United States of America

## Abstract

The human receptor for advanced glycation endproducts (RAGE) is a multiligand cell surface protein belonging to the immunoglobulin superfamily, and is involved in inflammatory and immune responses. Most importantly, RAGE is considered a receptor for HMGB1 and several S100 proteins, which are Damage-Associated Molecular Pattern molecules (DAMPs) released during tissue damage. In this study we show that the *Ager* gene coding for RAGE first appeared in mammals, and is closely related to other genes coding for cell adhesion molecules (CAMs) such as ALCAM, BCAM and MCAM that appeared earlier during metazoan evolution. RAGE is expressed at very low levels in most cells, but when expressed at high levels, it mediates cell adhesion to extracellular matrix components and to other cells through homophilic interactions. Our results suggest that RAGE evolved from a family of CAMs, and might still act as an adhesion molecule, in particular in the lung where it is highly expressed or under pathological conditions characterized by an increase of its protein levels.

## Introduction

The receptor of advanced glycation endproducts (RAGE) is a transmembrane protein belonging to the immunoglobulin (Ig) superfamily, and after signal peptide cleavage is composed of an extracellular domain containing three Ig-like domains, a single transmembrane helix and a cytoplasmic tail [1]. RAGE acts as a pattern recognition receptor (PRR) involved in inflammation resolution leading to tissue repair or alternatively in its perpetuation leading to chronic inflammation [2]. RAGE binds a large variety of molecules, including the so called advanced glycation endproducts (AGEs) that give it its name. RAGE is also a receptor for Damaged-Associated Molecular Pattern molecules that originate from damaged cells and alert the immune system to tissue trauma [3]. In particular, RAGE interacts with high mobility group box 1 (HMGB1), the prototypical DAMP, and S100 proteins [4]. How RAGE can interact with a diverse variety of molecules has been discussed by one of us in a recent review [5].

RAGE appears to be involved in many different disease states, including cancer [6], retinal disease [7], atherosclerosis and cardiovascular disease [8], Alzheimer’s disease [9], respiratory disorders [10], liver disease [11], and diabetic nephropathy [12]. Mice lacking RAGE are viable and apparently healthy, and appear to be resistant to many of the disease states listed above [13] [14]. This suggests that RAGE might be an effective and safe target to treat many different diseases. Yet, RAGE has several characteristics that set it apart from other receptors. RAGE appears to be multimerized before ligand binding [15]. Moreover, its best characterized interactor on the intracellular side is Diapahanous-1 (Dia-1), a cytoskeletal protein [1]. Finally, RAGE is expressed at very low levels in a number of cell types [16], as would be expected from a receptor, but is expressed at extremely high levels in normal lung [17], and specifically in alveolar type I (AT-I) cells [18], implying the possibility that RAGE might have a function in lung that is different from its function in other cells.

To better understand the function(s) of RAGE, we analyzed its evolutionary origin. Our data indicate that RAGE first appeared in mammals, and is closely related to adhesion molecules considering amino acid sequence and 3D structure. Indeed, when RAGE is forcibly expressed in cells that exhibit no expression, it endows them with the ability to adhere to components of the extracellular matrix and to other cells through homophilic interactions. Our results suggest that RAGE derived from an adhesion molecule, and might still have this function in the lung and possibly in pathological contexts.

## Materials and Methods

### Sequence Analysis

All protein sequence analyses have been performed using: protein-protein BLAST (BLASTp: http://www.ncbi.nlm.nih.gov/BLAST, [19]; the CLUSTALW multiple sequence alignment program (http://www.ebi.ac.uk/Tools/msa/clustalo/, [20]). Genome sequence analyses have been performed using the University of California Santa Cruz (UCSC) BLAT Search Genome (http://genome.ucsc.edu, [21]). EggNOG v. 3.0 [22] has been used in order to assign the origin of the genes. EggNOG database (http://eggnog.embl.de) contains orthologous groups constructed from more than one thousand organisms. For each orthologous group a phylogenetic tree is also provided; manual inspection of the trees allows us to assign the origin of the analysed genes to the most ancient node in the tree.

### Database Search

The search for proteins with high structural similarity to RAGE was performed using the DALI server [23]. The coordinates of the Ig domains of RAGE single V (residues 23–119), C1 domain (residues 120–236), C2 domain (residues 228–323) (pdb code 2ENS), and tandem Ig domain V-C1 (residues 23–232) (pdb codes 3CJJ, 3O3U) [24], [25] were used as query protein structures. For each set of coordinates the first 500 structural neighbours computed by DALI were inspected. DALI generated multiple sequence alignments were read into Clustal X [26], [27] to generate a phylogentic tree, which was analyzed using TreeView [28].

### Model Generation and Comparison

A model for the two N-terminal Ig domains of human MCAM was derived from the 3D modeling Server I-TASSER [29], [30]. A 3D model for the two N-terminal Ig domains of human ALCAM was created with the software MODELLER [31], [32] using the structures of RAGE V-C1 domains as template. Figures were prepared using PyMol [33].

### Recombinant Soluble RAGE Production and Surface Plasmon Resonance

Human recombinant sRAGE (aa 23–327) was expressed and purified as described previously [34]. sRAGE was cloned into vector pET15b and expressed in *Escherichia coli* BL21(DE3) Origami B with an N-terminal His_6_-tag. Cells were grown in shaking culture in DYT medium supplemented with 50 mM sodium phosphate, 0.2% glucose, and 100 µg/ml ampicillin at 37°C to an OD_600 nm_ = 0.6. Then the temperature was shifted to 23°C and expression was induced by the addition of 0.5 mM IPTG at OD_600 nm_ = 1. The culture was grown for 12 h, then chilled on ice and cells were harvested by centrifugation. Typically, 10 g wet weight cells were resuspended in 20 ml of 50 mM sodium phosphate, 30 mM imidazole, pH 7.4, containing protease inhibitors (Complete; Roche) and were ruptured by two passages through a French pressure cell. The cell lysate was subjected to ultracentrifugation at 100,000 g for 1 h and the supernatant was diluted 5-fold in 50 mM sodium phosphate, 300 mM NaCl, 30 mM imidazole, pH 7.4 and applied to Ni-Sepharose Fast Flow column (GE-Healthcare, UK) equilibrated in the same buffer. Proteins without His_6_-tag were washed out with the same buffer and bound sRAGE was eluted in a linear gradient over five column volumes with 50 mM sodium phosphate, 300 mM NaCl, 500 mM imidazole, pH 7.4. sRAGE was concentrated by ultrafiltration and applied to Superdex 75 column (26/600, GE Healthcare) equilibrated in 20 mM HEPES, 300 mM NaCl, pH 7.5 and eluted in the same buffer. sRAGE without His_6_-tag was obtained by thrombin (GE Healthcare) cleavage. Prior cleavage the protein was dialyzed against 20 mM Tris–HCl, 300 mMNaCl, 2 mM CaCl_2_, pH 8.0. Thrombin was added (0.2 U per mg of protein) and sRAGE was incubated at room temperature for 1–2 hr. Protein still containing the His_6_-tag was removed by a separation on a MonoS column (GE Healthcare) equilibrated in 20 mM sodium acetate, pH 5.0, applying a linear gradient of 20 mM sodium acetate, 1 M NaCl, pH 5.0 over 20 column volumes.

Surface plasmon resonance protein-protein interaction analysis was performed on a Biacore X instrument. sRAGE carrying a His_6_-tag at the N-terminus was added to a final concentration of 150 nM to the running buffer (in 20 mM HEPES, 150 mM NaCl, 0.005% P20, pH 7.6) and immobilized on Ni-NTA-chip at a flow rate of 10 µl/min for 1 min. Typically 1500–2000 RU of protein was immobilized on the sensor chip. sRAGE carrying no His_6_-tag was diluted in running buffer and the binding was analyzed at 298 K. Data were fitted using BIAevaluation software version 4.1 (BIACORE - GE Healthcare).

### Generation of RAGE Expression Plasmids

In order to construct pcDNA-neo-FL-RAGE or p-LXSN-neo-FL-RAGE vectors, the human FL-RAGE cDNA (GenBank2 accession no. NM001136) was excised from a pre-existing vector by using EcoRI and XhoI restriction endonucleases (New England Biolabs, MA, USA) and inserted into pcDNA-3 or pLXSN (Clontech, CA, USA) digested with same enzymes. To generate pCAGS-IRES-GFP-FL-RAGE, human FL-RAGE cDNA was excised with XhoI, filled-in to create blunt ends, and subsequentially digested with EcoRI. The purified cDNA was then inserted in pCAGS-IRES-GFP (kindly provided by Vania Broccoli) cut with EcoRI and SmaI (Promega, USA).

### Cell Culture, Transfection and Generation of Stable Lines Expressing RAGE

Murine preB 300.19 cells were grown in RPMI 1640 medium supplemented with 10% fetal calf serum (FCS), 1% penicillin/streptomycin and 50 µM β-mercaptoethanol. HEK293 and R3/1 (kindly provided by Roland Koslowski and Michael Kasper) cells were grown in Dulbecco modified Eagle medium (DMEM) supplemented with 10% FCS and 1% penicillin/streptomycin. PreB 300.19 cells were transfected with pCAGS-IRES-GFP or pCAGS-IRES-GFP-FL-RAGE vectors to generate preB/CAGS or preB/FL-RAGE respectively, using Lipofectamine according to the manufacturer’s instructions (Invitrogen, Carsbad, CA, USA). Transfectants were sorted using GFP as marker and positive cells were grown. Photographs were taken at 20× magnification in phase contrast (Leica DM IRB). To generate HEK-pcDNA or HEK/FL-RAGE cells, HEK293 cells were transfected with pcDNA-neo or pcDNA-neo-FL-RAGE using Fugene 6 (Roche Molecular Biochemicals, Mannheim, Germany) according to the manufacturer’s instructions. Cells were selected with 500 µg/ml G418 (Invitrogen). To generate R3/1-pLXSN or R3/1-FL-RAGE clones, R3/1 cells were infected with retrovirus carrying p-LXSN-neo or p-LXSN-neo-FL-RAGE retro-vectors. Clones were selected with 500 µg/ml G418 (Invitrogen).

### Western Blot (WB)

Cells were analyzed for human FL-RAGE expression by WB as already described [35] using a goat polyclonal antibody against the extracellular domain of human RAGE (α-RAGE N-term1, 1 µg/ml; cat. AF1145, R&D Systems, Minneapolis, MN, USA) that recognizes rat RAGE as well. For detection of RAGE in rat lung and R3/1 cells lysates two additional different rabbit polyclonal antibodies against the extracellular (α-RAGE N-term2, 0.4 µg/ml; cat. sc-5563, Santa Cruz Biotechnology, California, USA) and the intracellular (α-RAGE C-term, 1 µg/ml; cat. ab3611, Abcam, Cambridge, UK) domains of human RAGE were used. Both antibodies recognize rat RAGE as well.

The membranes were blocked in TBST (10 mM Tris, pH 7.4; 0.5 mM NaCl; 0.1% Tween 20) containing 5% powdered skimmed milk for 1 hour (h) at rt. The blots were first probed with the indicated primary antibodies diluted in TBST with 5% powdered skimmed milk over night at 4°C, and then with horseradish peroxidase-conjugated anti-rabbit (1∶5000; cat. NA9340V, GE Healthcare) or anti-goat (1∶5000; cat. sc-2020, Santa Cruz Biotechnology) secondary antibodies. Proteins were visualized by an enhanced chemiluminescence (ECL) detection system (cat. RPN2106, GE Healthcare). An antibody agaist GAPDH (0.4 µg/ml; cat. sc-25778, Santa Cruz Biotechnology) was used on the same membranes after stripping and served as loading control.

### Cell-cell, Cell-matrix Adhesion and Cell Spreading Assays

#### Cell-matrix adhesion and cell spreading

The assays were performed as described previously with following modifications [17]. Tissue culture plates were coated for 2 hours at 37°C with 10 µg/ml collagen I (C7661), laminin (L2020), fibronectin (all from Sigma, St. Louis, MO, USA) or PBS. For cell adhesion assay, after saturation of wells with DMEM +1% BSA, R3/1-pLXSN or R3/1-FL-RAGE cells (5×10^4^) were resuspended in DMEM +10% FCS and seeded on coated 96-well plates in triplicate for 15 or 45 minutes at 37°C under 5% CO_2_. Cells were extensively washed with PBS, fixed with 4% paraformaldehyde, stained with crystal violet for 10 min, lysed with 2% SDS and read on an ELISA plate reader at 550 nm. For cell spreading assay, R3/1-pLXSN or R3/1-FL-RAGE cells (5×10^4^) were resuspended in DMEM +10% FCS and seeded on 12-well coated plates in triplicate for 90 minutes at 37°C under 5% CO_2_. Cells were then washed with PBS and fixed with 4% paraformaldehyde. Photographs were taken at 40× magnification in phase contrast (Leica DM IRB). Cell surface area (µm^2^, 25–50 cells) was quantified using Axiovision Software™ Rel 4.7 (Zeiss).

#### Cell-cell adhesion

HEK-pcDNA or HEK/FL-RAGE cells were grown in six-well plates (Costar, Milan, Italy) for 24 hours. Confluent monolayers were washed with PBS and mechanically dissociated by pipetting 30 times as previously described [36]. Cells were photographed at 40× magnification in phase contrast (Leica DM IRB), and the number of particles (cell clusters) was determined (Np). An aliquot of the same cell suspension was trypsinized, and the number of single cells was determined (Nc). The dissociation index was expressed as Np/Nc.

#### Cell aggregation assay

PreB/pCAGS or preB/FL-RAGE cells were seeded and cultured for the indicated hours before being photographed at 20× magnification in phase contrast with an Apotome microscope (Zeiss, Germany). Aggregates area of 3 different fields was measured with Axiovision Software™ Rel 4.7 (Zeiss). For “mixed” aggregation assays, a double colored assay was performed as already described with same modifications [37], [38]. Briefly, preB cells or preB/FL-RAGE (that express GFP) were labelled fluorescent red with 5 µM of CellTracker™ Orange CMTMR (5-)and-6)-(((4-chloromethyl)benzoyl)amino) tetramethylrhodamine (Invitrogen) in RPMI 1640 medium for 45 min at 37°C. After extensive washing, cell lines were cultured as single cell suspensions or mixed in equal number (5×10^4^) as indicated and allowed to aggregate in growth medium for 24 h at 37°C under 5% CO_2_. Images were taken using an Apotome microscope (Zeiss, Germany) with a 20× objective and Axiovision Software™ Rel 4.7 (Zeiss).

### Immunofluorescence (IF)

For IF analysis, HEK-pcDNA, HEK/FL-RAGE cells or a mix of equal number of both cell types (1×10^5^ total number) were seeded on glass coverslips and after two days of culturing, fixed in 4% paraformaldehyde at room temperature (RT) for 30 min. Cells were then washed by PBS followed with 1 h blocking in PBS+5% donkey serum (Sigma). After washing 3 times with 1% donkey serum plus 0.1% Triton (DST), cells were incubated for 1 h at rt with goat anti-human RAGE antibody (4 µg/ml; cat. AF1145, R&D Systems) in DST buffer. Cells were then incubated for 1 h at rt with donkey anti-goat conjugated with Alexa Fluor 546 (1∶1000; Invitrogen, USA). Nuclei were couterstained with Hoechst 33342 (1∶2000; Invitrogen) for 10 min at RT. Coverslips were mounted with Dako Fluorescent mounting medium (DAKO, CA, USA). Images were taken using an Apotome microscope (Zeiss, Germany) with a 20× objective and Axiovision Software™ Rel 4.7 (Zeiss). A similar analysis was performed on R3/1-pLXSN or R3/1-FL-RAGE cells avoiding addition of 0.1% Triton during washes.

### Statistical Analysis

Data were analyzed by two-tailed Student’s *t*-test and differences were considered statistically significant when *P*≤0.05.

## Results and Discussion

### Phylogenetic Analysis of the *Ager* Gene

In order to identify *Ager* genes in other Eukaryotes, we performed a BLASTp search using the human RAGE protein sequence as query. *Ager* genes are present in Primates, Glires and Laurasiatheria, while we found no orthologous genes in genomes of other eukaryotic model organisms. Moreover, using BLAT Search Genome analyses, we scanned the complete genomes of model organisms for each clade and we did not find any sequence matching with the human *AGER*.

**Figure 1:**
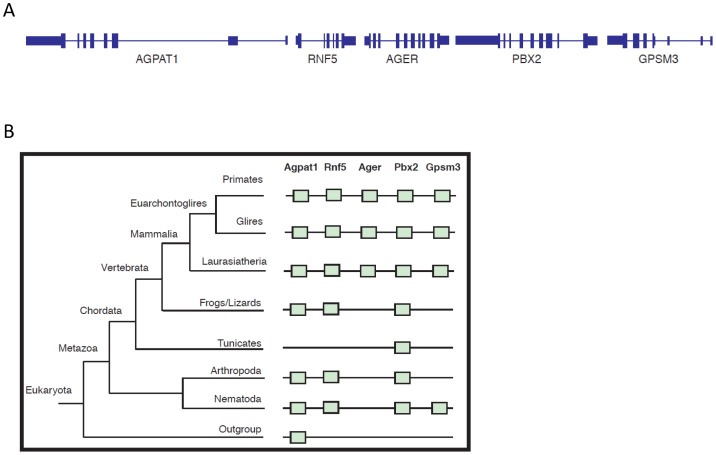
Phylogenetic analysis of the *Agpat1*–*Gpsm3* locus including *Ager*. (**A**) The panel represents the structures of the *Agpat1*-*Rnf5*-*Ager*-*Pbx2*-*Gpsm3* genes. The locus spans more than 1 Mbp in human chr 6. *Ager* (receptor for advanced glycation endproducts); *Agpa1* (1-acylglycerol-3-phosphate O-acyltransferase 1); *Gpsm3* (G-protein signaling modulator 3); *Pbx2* (pre-B-cell leukemia homeobox 2); *Rnf5* (ring finger protein 5). (**B**) A schematic phylogenetic tree with the main nodes. The presence of a gene is depicted by a green box, while the absence of the box indicates that the gene is not found in the specific phylogenetic group.

To exclude the possibility that this could be due to incomplete sequencing or lacking of annotation information of the genomes, we repeated the searches using the genomic locus containing *Ager* plus two genes on the telomeric side, *Agpat1* and *Rnf5,* and two on the centromeric side, *Pbx2* and *Gpsm3* (Fig. 1A). In order to date the appearance in evolution of the genes in the genomic locus, we used the application EggNOG v3.0. A schematic representation of the results obtained is shown in Fig. 1B. The analyses reveal that *Laurasiatheria, Glires* and *Primates* show perfect synteny of the investigated genes; earlier in evolution, a syntenic locus containing putative *Agpat1*, *Rnf5*, *Pbx2* and *Gpsm3* genes, but not *Ager*, was found in Metazoans. *Gpsm3* appears with Metazoans, and is present in Mammalia, but it is absent in Frogs and Chordata, possibly because of secondary loss.

These results suggest that *Ager* first appeared in the genomic region between the *Rnf5* and *Pbx2* genes during the radiation of mammals, and the syntenic locus itself first appeared with Eukaryotes. Apparently, *Ager* was secondarily lost in *Aves*. The *Agpat–Gpsm3* locus lacks highly conserved intergenic sequences, suggesting that genetic control elements are rare or are subject to rapid evolution. In both cases, the *de novo* appearance of the *Ager* gene in the locus could have been readily tolerated.

### *Ager*, *Alcam*, *Mcam*, *Bcam* Genes Share a Common Ancestor

In order to reconstruct the origin of the *Ager* gene, we looked for proteins sharing sequence identities with the human RAGE. A BLASTp search (restricted to *H. sapiens*) revealed a weak sequence identity (E value = 8×10^−14^) to the Activated Leukocyte Cell Adhesion Molecule (ALCAM/CD166). We then performed a BLASTp search in the human proteome using the ALCAM protein as query. In addition to RAGE, we identified two other cell adhesion molecule proteins belonging to the Ig superfamily with significant sequence identities: Lutheran blood group antigen/Basal-Cell Adhesion Molecule (Lu/BCAM) (E value = 4×10^−38^) and Melanoma Cell Adhesion Molecule (MCAM/CD146/MUC-18/gicerin) (E value = 10^−43^).

**Figure 2:**
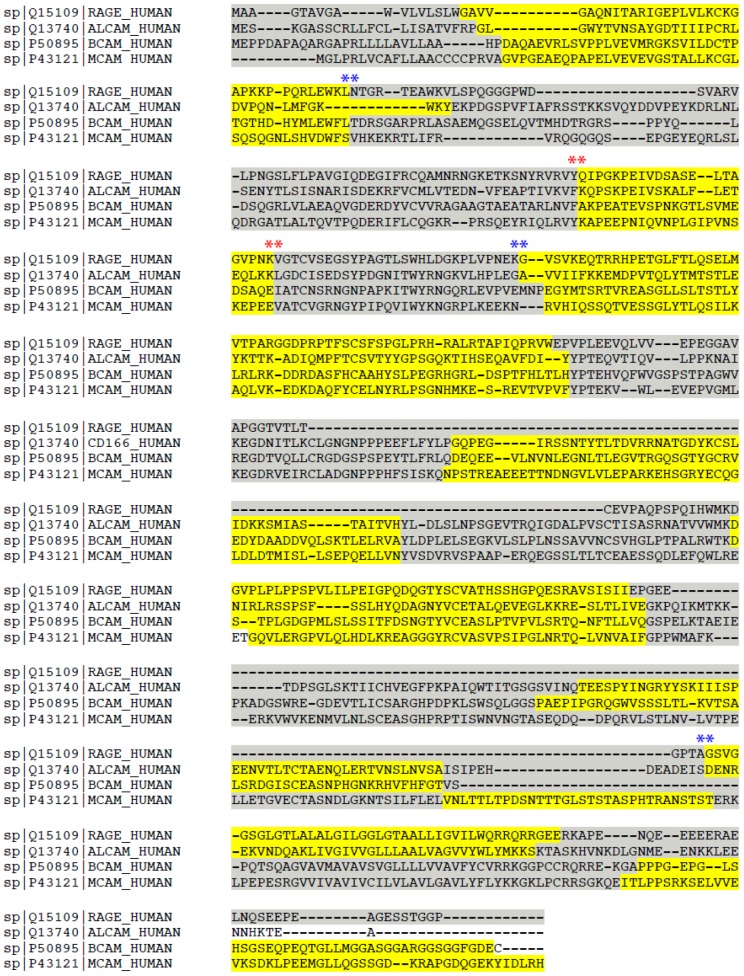
Comparison of the RAGE, ALCAM, BCAM and MCAM protein sequences. The multiple alignment was performed with CLUSTALW. Exons encoding a portion of the corresponding protein are highlighted alternatively in yellow and grey. Asterisks indicate conserved exon-intron boundaries; if conservation is among all four proteins, the asterisks are red, if conservation is between RAGE and at least one of the others the colour is blue. RAGE (receptor for advanced glycation endproducts); ALCAM (activated leukocyte cell adhesion molecule); BCAM (basal cell adhesion molecule); MCAM (melanoma cell adhesion molecule).

We then compared the genomic organization of the four genes coding for RAGE, ALCAM, BCAM and MCAM. Interestingly, aligning the protein sequences and the exon-intron boundaries (Fig. 2), we found several boundaries that are highly conserved at least in three of them, suggesting that these genes probably appeared due to duplication events of a common ancestor gene.

The conservation of the boundaries occurs particularly in those gene segments encoding for extracellular Ig-like domains; RAGE and ALCAM shared also the same genomic organization in the gene fragment coding for the cytoplasmic domain.

### RAGE is Structurally Closely Related to CAMs

A 3D structural search can often reveal evolutionary relationships which are not or only barely detectable on the basis of sequence information. We therefore subjected the Ig domains of RAGE V, C1, and C2 as well as V-C1 tandem domains to protein structure database search using the DALI server. Criteria for a successful alignment are the RMSD of the aligned structures, the length of the alignment and the match of secondary structure elements. DALI additionally evaluates the similarity of two structures by comparing intramolecular distances [39].

Searching the PDB database with the single V domain, C1 domain, or C2 domain of RAGE as query structures resulted in a hit list with a wide range of different molecules. Such a result was not unexpected since the fold of the different subsets of Ig domains is very well conserved throughout the entire Ig superfamily [40], [41]. The vast majority of Ig domains similar to those of RAGE derived from antibodies, which account for the largest group of Ig molecules where structural information is available. Only 4 out of 500 similar structures were from cell adhesion molecules or cell surface receptors. In stark contrast, when we analysed the results of a database search with RAGE V-C1 tandem domain, we found a number of hits from cell adhesion molecules which mediate homophilic cell-cell contacts. After removal of duplicates and short alignments with less than 150 residues pairs, we obtained 24 different homologous cell adhesion molecules, 2 cell surface receptors, and only one antibody light chain as closest structural homologues among the 500 best hits. The two cell surface receptors related are T-cell receptor αβ type and T-cell receptor δγ type. By far the best structural alignment was observed between the two N-terminal domains of human BCAM (pdb code 2PET) [42] and RAGE V-C1. The alignment of 194 residues showing an RMSD of 3.3 Å and the high DALI Z-score of 16.7 document very well the high similarity between both structures. Low RMSDs ranging from 2.7 to 5.4 Å were observed throughout in structural alignments of RAGE with the different cell adhesion molecules.

The results suggest that the spatial arrangement of V and C1 in RAGE is very well conserved and resembles closely those of other CAMs. In line with these results our previous analysis of the RAGE ectodomain structure by NMR and X-ray crystallography revealed that V and C1 form an integrated structural unit where both Ig domains form a bent elongated structure with an angle of 145 degrees between the two Ig domains [5], [43]. Inspection of the cell adhesion molecules retrieved from the DALI database search showed that the corresponding Ig domains exhibit a similar curved structure.

**Figure 3:**
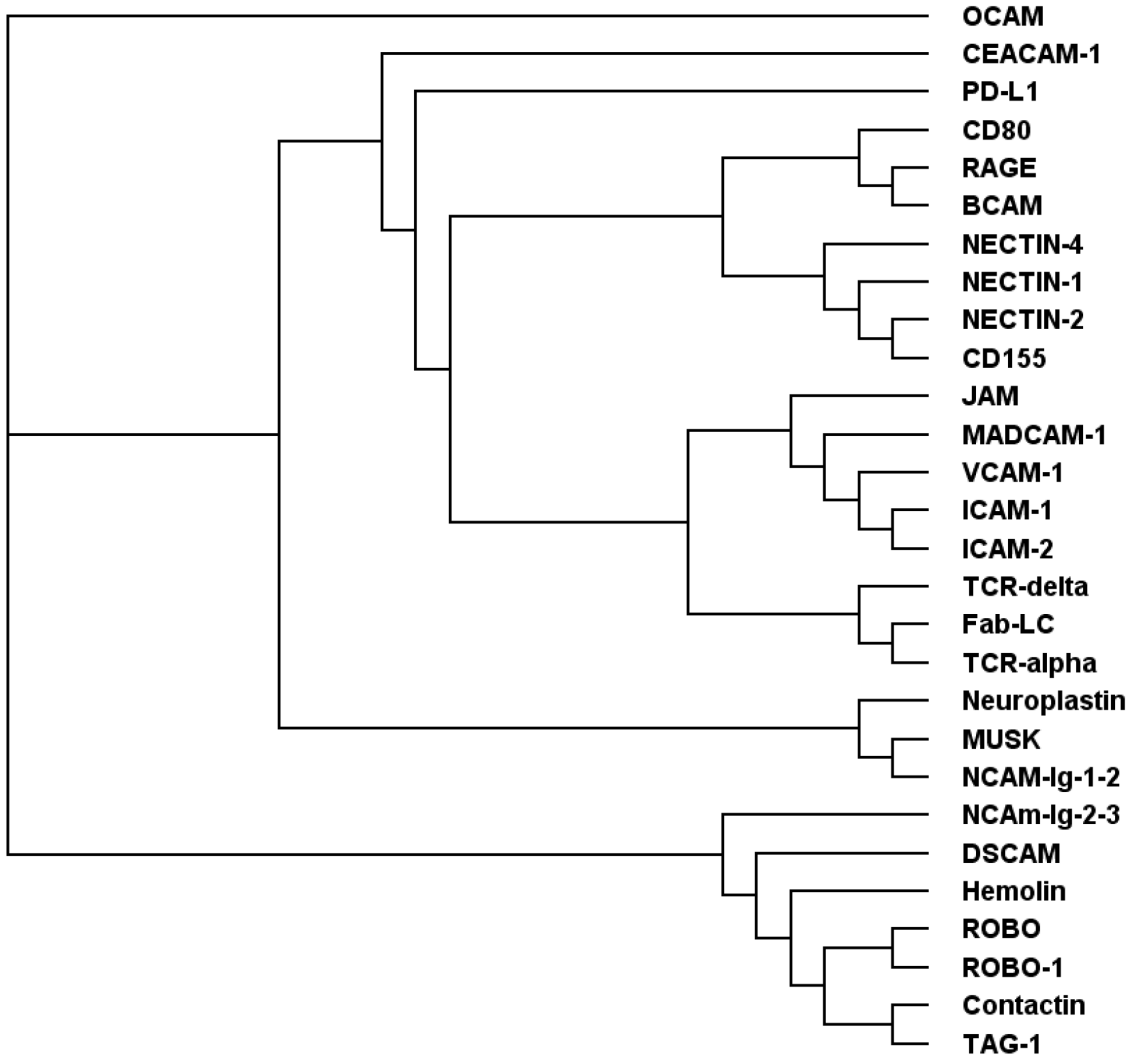
Phylogenetic tree derived from 3D structural alignments. The phylogenetic tree is based on the analysis of the closest 500 matches resulting from a search of the protein structure database using the structure of RAGE Ig domains V-C1 as a query. After filtering the results for a minimum length of alignment and removal of duplicates 27 different proteins were retrieved. Twenty four out of these 27 are cell adhesion molecules, only 2 belong to the T-cell receptor family and one protein represents the light chain of an antibody. RAGE V-C1 Ig domains group very closely with cell adhesion molecules BCAM and CD80. OCAM (olfactory cell adhesion molecule; pdb code 2jll); CEACAM-1 (Carcinoembryonic antigen-related cell adhesion molecule 1, CD 66a; pdb code 3R4D); PD-L1 (programmed death1 inhibitory receptor; pdb code 3BIS); CD80 (cluster of differentiation 80; pdb code 1DR9); RAGE (receptor for advanced glycation endproducts; pdb code 3CJJ); BCAM (Lutheran glycoprotein; basal cell adhesion molecule; pdb code 2PET); NECTIN-1 (pdb code 4FMF); NECTIN-2 (pdb code 4FMK); NECTIN-4 (pdb code 4FRW); CD155 (cluster of differentiation 155, poliovirus receptor; pdb code 3URO); CD2 (cluster of differentiation 2; pdb code 1HNG); MADCAM-1 (mucosal vascular addressin cell adhesion molecule 1; pdb code 1GSM); VCAM (vascular cell adhesion molecule; pdb code 1VSC); ICAM-1 (intercellular adhesion protein 1, pdb code 1IC1); ICAM-2 (intercellular adhesion protein 2; pdb code 1ZXQ); TCR-alpha (T-cell receptor a chain; pdb code 1NFD); TCR-gamma (T-cell receptor g chain; pdb code 1HXM); Fab-LC (antibody Fab fragment light chain; pdb code 3QNX); Neuroplastin (pdb code 2WV3); MUSK (pdb code 2IEP); NCAM-Ig-1–2 (neural cell adhesion molecule Ig domains 1 and 2; pdb code 1EPF); NCAM-Ig-2–3 (neural cell adhesion molecule Ig domains 1 and 2; pdb code 1QZ1); DSCAM (Down syndrom cell adhesion molecule; pdb code 3DMK); Hemolin (pdb code 1BIH); ROBO (Roundabout; pdb code 2VRA); ROBO-1 (Roundabout homolog 1; pdb code 2V9R); Contactin (protein tyrosine phosphatase z (PTPRZ); pdb code 3JXA); TAG-1 (axonin, pdb code 1CS6).

In order to classify RAGE further, we used the DALI search results to generate a structure-based multiple sequence alignment that was basis for a phylogenetic tree (Fig. 3). In this tree RAGE clusters closely with BCAM and CD80 (pdb code 1DR9) as well as with a branch that comprises the Nectins (pdb codes 4FMF, 4FMK, 4FRW, CD155 (pdb code 3URO). A further cluster is formed by the cell adhesion molecules JAM (junctional adehsion molecule; pdb code 1NBQ), MADCAM-1 (mucosal vascular addressin cell adhesion molecule 1; pdb code 1GSM), VCAM (vascular cell adhesion molecule; pdb code 1VSC), ICAM-1 (intercellular adhesion protein 1, pdb code 1IC1), ICAM-2 (intercellular adhesion protein 2, pdb code 1ZXQ). The branch most distant from RAGE contains cell adhesion molecules from non-mammalian species, such as hemolin (pdb code 1BIH) from *Hyalophora cecropia* (moth), ROBO (pdb code 2VRA) from *Drosophila*, and TAG-1 (pdb code 1CS6) from *Gallus gallus,* but also the mammalian ortholog to ROBO, the so-called ROBO-1 (pdb code 2VR9). Interestingly, despite the large phylogenetic distances between these species, the basic structural features required for homophilic or heterophilic interaction are well conserved. The observed arrangement of the Ig domains might represent a core structure that is favourable for intermolecular interactions but varied and fine-tuned for each specific case.

**Figure 4:**
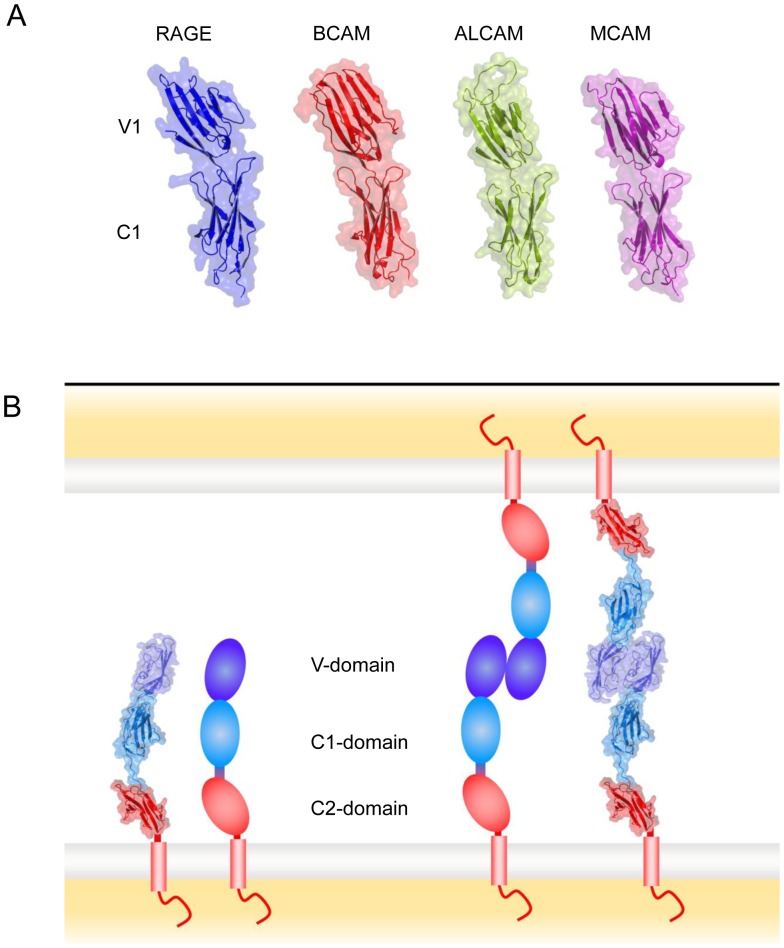
Comparison of RAGE V-C1 structure with homophilic cell adhesion molecules BCAM, ALCAM and MCAM. (**A**) The Ig domains V and C1 of RAGE which reside most distal from the cytoplasmic membrane (shown in blue) adopt a slightly bent structure. This spatial arrangement is very well conserved in the Ig domains 1 and 2 of its close homologue BCAM (red). Structural models of the corresponding Ig domains of ALCAM (green) and MCAM (magenta) suggest that these adopt as well a very similar structure that might be required for homophilic interaction. RAGE (receptor for advanced glycation endproducts); ALCAM (activated leukocyte cell adhesion molecule); BCAM (basal cell adhesion molecule); MCAM (melanoma cell adhesion molecule). (**B**) Model for RAGE-RAGE homophilic interaction mediating cell adhesion (derived from pdb code 4LP5) [44]. Left hand side: as observed in the crystal structure of sRAGE the extracellular domain adopts an extended conformation; alongside a cartoon representation is shown. Right hand side: in the crystal the V-domains (dark blue) form a large contact in trans orientation. This mode of interaction is conserved among different crystal structures suggesting that RAGE homophlic interaction occurs via the V-domain.

In order to illustrate the structural similarity between the phylogenetically related RAGE, BCAM, ALCAM and MCAM we prepared structural models for ALCAM and MCAM depicted in Fig. 4A. The models for ALCAM and MCAM exhibit like RAGE V-C1 or BCAM Ig-1–2 that the two Ig domains adopt a slightly bent structure as a characteristic feature.

In summary, the DALI search results show clearly that RAGE is classified structurally as a cell adhesion molecule mediating homo- or/and heterophilic interactions. The similarity of RAGE V-C1 to BCAM Ig-1–2 is striking, and structural modelling suggests that this similarity is conserved as well in ALCAM and MCAM Ig domains 1 and 2.

### Putative Mode of Homophilic Interaction

Recent X-ray crystallographic studies strongly support homophilic interaction of RAGE and suggest that binding occurs via the V-domain. A trans-orientated interaction of RAGE (Fig. 4B) is inferred from the arrangement of sRAGE or RAGE VC1 domains in different crystal structures where the V-domains from both molecules form a large hydrophobic contact. This arrangement appears to be highly conserved among the different crystal structures and is observed for single VC1 (pdb code 3CJJ, 4LP4) [24], [44] or sRAGE (pdb code 4LP5) [44] or for VC1 in complex with heparan sulfate (pdb code 4IM8) [45] or with DNA (pdb codes 3S58, 3S59) [46]. Virtually no structural change is observed in this dimeric assembly between unbound RAGE or RAGE engaged with heparan sulfate or DNA as ligand suggesting that this interaction is rather stable already in the absence of a ligand corroborating the proposed function as a cell adhesion molecule.

### RAGE Enhances Cell-matrix and Cell-cell Adhesion

**Figure 5:**
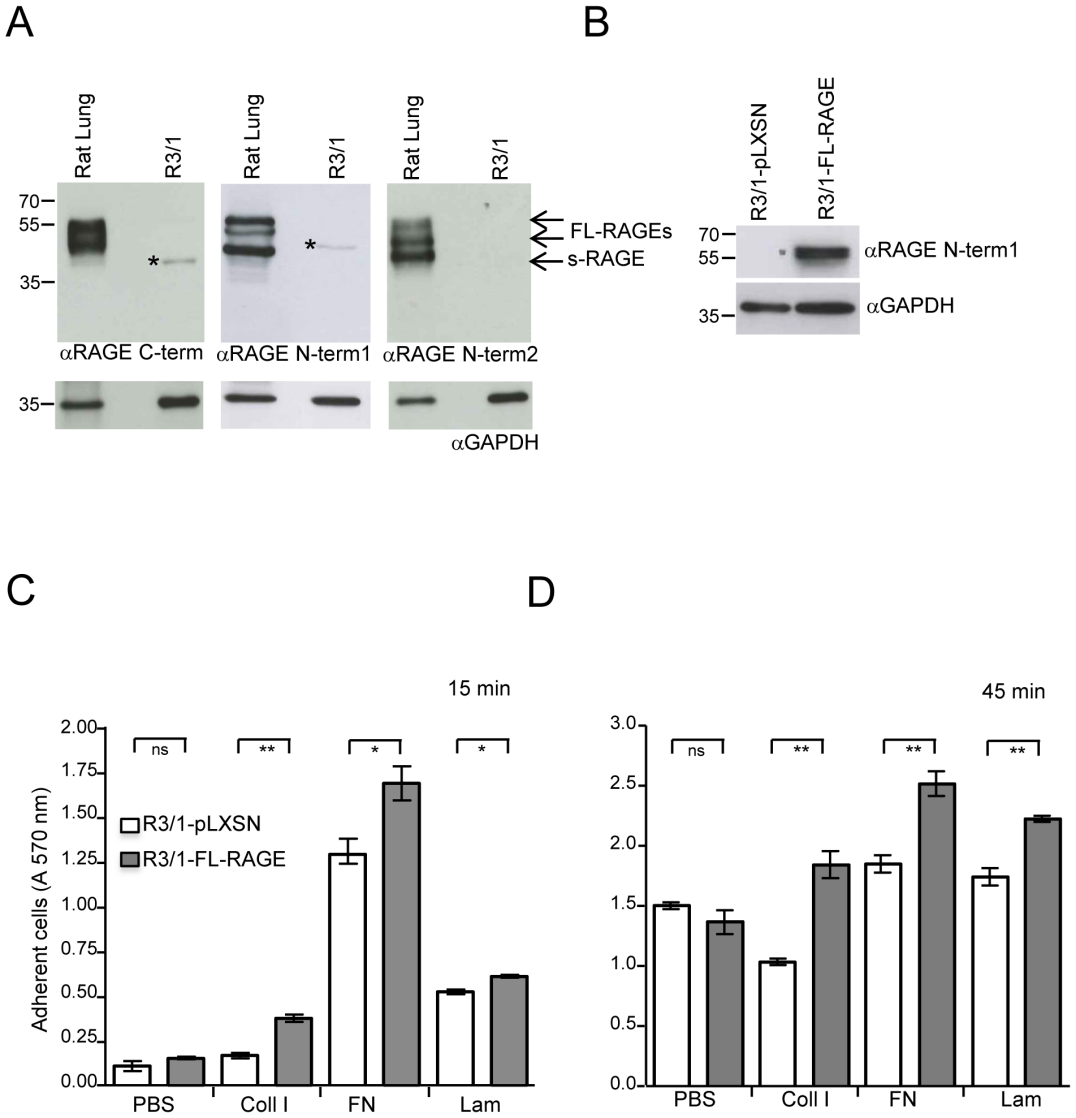
RAGE expression enhances cell-matrix adhesion. (**A**) Western blot analysis on rat lung lysate and R3/1 cells using three different antibodies recognizing RAGE extracellular (anti-RAGE N-term1 and anti-RAGE N-term2) or intracellular (anti-RAGE C-term) domains. Asterisk (*) indicates nonspecific bands. Forty µg of cell lysate were loaded and detection of GAPDH was used as loading control**.** (**B**) Western blot analysis on R3/1-pLXSN and R3/1-FL-RAGE cells using anti-RAGE N-term1 antibody. Forty µg of cell lysate were loaded and detection of GAPDH was used as loading control**.** (**C, D**) Cell-matrix adhesion assay. Adhesion of R3/1-pLXSN or R3/1-FL-RAGE cells onto culture dishes coated with 10 µg/ml ECM proteins. Adhesion to PBS, collagen I (Coll I), Fibronectin (FN), or Laminin (Lam) was assayed for 15 minutes (C) or 45 minutes (D). One representative experiment out of three is shown. Results from triplicate wells are displayed as means±SEM (ns, not significant; *, P<0.05; **, P<0.01).

**Figure 6:**
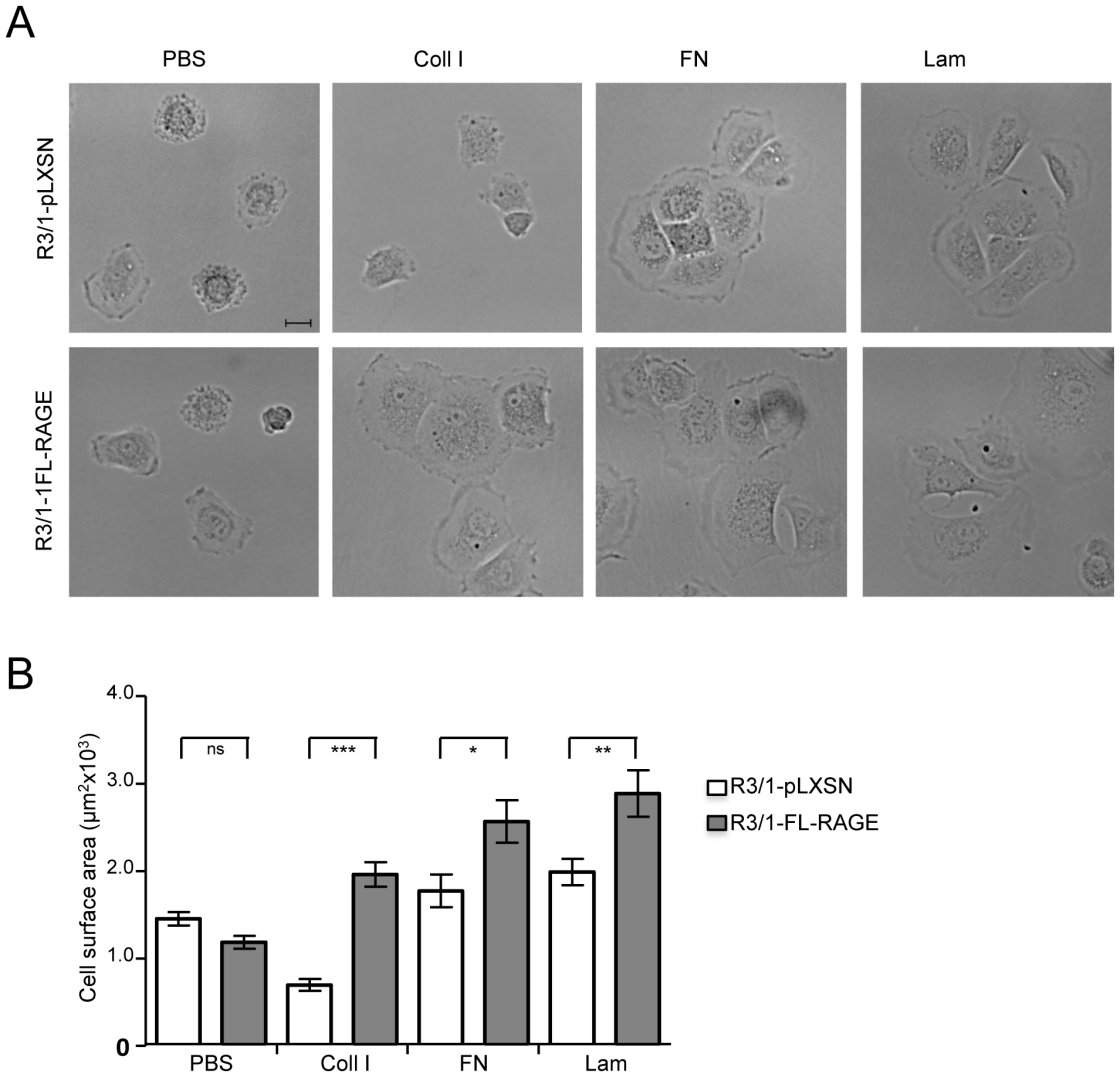
RAGE expression enhances cell spreading. Cell spreading assay. (**A**) Spreading of R3/1-pLXSN or R3/1-FL-RAGE cells onto culture dishes coated with 10 µg/ml of ECM proteins (Coll I, FN, or Lam) or PBS was assessed at 90 minutes after seeding. Photographs were taken in phase contrast at 40× magnification. Bar corresponds to 20 µm. (**B**) Quantification of cell spreading based on cell surface area. Results are displayed as means±SEM (ns, not significant; *, P<0.05; **, P<0.01; ***, P<0.001).

In order to address the question whether RAGE has adhesive properties and to experimentally verify the phylogenetic and structural predictions, we performed several adhesion assays. RAGE has already been shown to promote adherence and induce cell spreading to extracellular matrix (ECM) components in HEK and A549 cells [17], [47] and to AGE-modified components of the ECM of prostate cancer cells [34]. RAGE is expressed at very low levels in most tissues, but at high levels in the lung [17], [48], [49] where it mainly localizes at the basal cell membrane of alveolar type I (ATI) cells and appears to be responsible for the flat morphology required for gas exchange [17], [18], [50]. Thus, we decided to test RAGE interactions with cell matrix components using the rat alveolar type I-like cell line R3/1, that displays several phenotypical features of alveolar epithelial type I cells [51]. By WB using three different antibodies we were not able to detect endogenous RAGE in R3/1 cells (Fig. 5A). Conversely, in rat lung tissue, three major RAGE isoforms were present; in particular, the lowest band represents soluble RAGE not detectable by the αRAGE C-term antibody (Fig. 5A). We generated stable clones of R3/1 cells expressing full-length RAGE cDNA (R3/1/FL-RAGE) or the empty vector (R3/1-pLXSN) as control (Fig. 5B). The adhesive capacity of R3/1 clones was time- and extracellular matrix (ECM) component-dependent, however, compared to R3/1-pLXSN, R3/1-FL-RAGE cells adhered faster and to a higher percentage to all tested ECM molecules, with the major difference observed for collagen I (Fig. 5C,D). Accordingly, the expression of RAGE enhanced spreading of adherent cells in a similar manner (Fig. 6). Thus, our results confirm previous studies that demonstrate heterophilic interactions between RAGE and ECM components.

**Figure 7:**
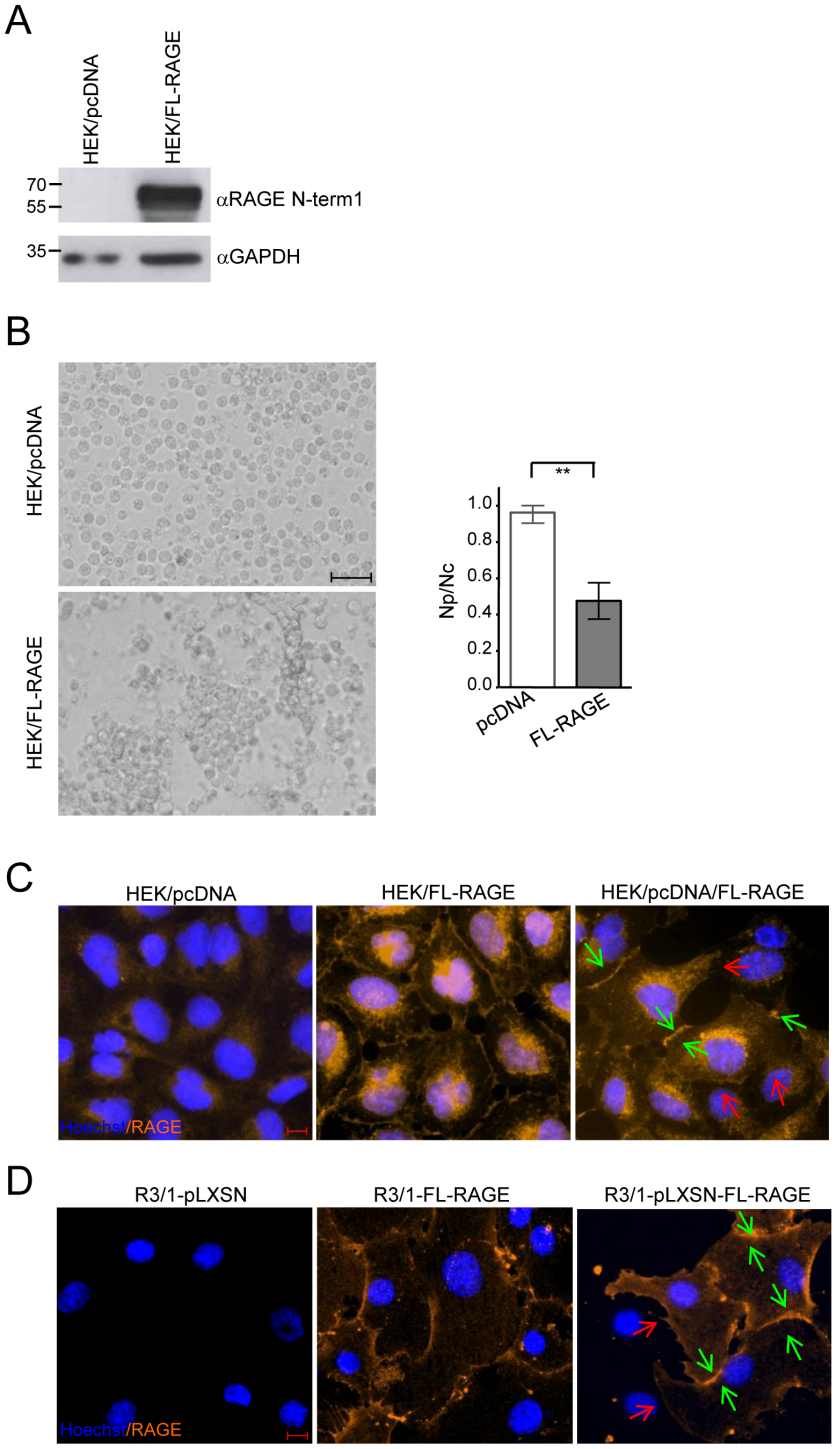
RAGE expression contributes to cell-cell adhesion. (**A**) Western blot analysis on control HEK cells (HEK/pcDNA) or expressing FL-RAGE (HEK/FL-RAGE) using anti-RAGE N-term1 antibody. Forty µg of cell lysate were loaded and detection of GAPDH was used as loading control**.** (**B**) A dissociation assay was performed by applying mechanical force on monolayers of HEK/pcDNA or HEK/FL-RAGE cells. Photographs were taken at 40× magnification in phase contrast. Bar corresponds to 50 µm. The dissociation index is the means±SEM of three independent experiments (**, P<0.01). (**C**) Localization of FL-RAGE on HEK/pcDNA, HEK/FL-RAGE or a mix of both cell lines (orange). Nuclei are stained in blue. Red arrows indicate HEK/pcDNA cells in contact with HEK/FL-RAGE. Green arrows indicate HEK/FL-RAGE cells in contact with each other. Bar corresponds to 10 µm. (**D**) Localization of FL-RAGE on R3/1-pLXSN, R3/1-FL-RAGE or a mix of both cell lines (orange). Nuclei are stained in blue. Red arrows indicate R3/1-pLXSN cells in contact with R3/1-FL-RAGE. Green arrows indicates R3/1-FL-RAGE cells in contact with each other. Red arrows indicate cells not expressing RAGE in contact with cells expressing RAGE. Bar corresponds to 10 µm.

To assess the ability of RAGE to mediate cell-cell adhesion we performed a dissociation assay [36] using HEK293 cells stably expressing FL-RAGE (HEK/FL-RAGE) or the corresponding empty vector (HEK/pcDNA) (Fig. 7A). In contrast to R3/1 cells, HEK293 cells are able to form a compact monolayer that can be detached from culture dishes by repeated pipetting avoiding the use of trypsin. As shown in Fig. 7B (left panel), when applying mechanical force to confluent monolayer of these cells, HEK/FL-RAGE formed large clusters while HEK/pcDNA were completely dissociated. This effect was quantified by counting the number of particles (Np) observed and the total number of cells (Nc) (Fig. 7B, right panel). The ratio Np/Nc is a measure of dissociation, and varies between 1, complete dissociation of cells, and 0, no dissociation [36].

We then tested the localization of RAGE by IF on confluent cultures of HEK/pcDNA, HEK/FL-RAGE or a mix of both cell lines (HEK/pcDNA/FL-RAGE). HEK/pcDNA cells displayed only diffused background staining for RAGE (Fig. 7C). HEK/FL-RAGE cells exhibited a prominent ER-Golgi staining, in addition to plasma membrane labelling. In particular, these cells displayed an intercellular zone of intense fluorescence for RAGE when contact each other (Fig. 7C). When HEK/pcDNA and HEK/FL-RAGE were mixed and grown together, intense RAGE staining was observed mostly at cell-cell contacts of adjacent HEK/FL-RAGE cells (green arrows), while low or even no staining appeared when HEK/FL-RAGE where in contact with HEK/pcDNA cells (red arrows) (Fig. 7C). Similar results were obtained for R3/1 cells (Fig. 7D).

All together these data show that RAGE is highly enriched in zones mediating cell-cell contacts of adjacent cells. Moreover, the occurrence of RAGE in this areas suggests that it regulates cell-cell adhesion through homophilic interaction.

### RAGE Displays High Affinity Homophilic Interaction *in vitro*

**Figure 8:**
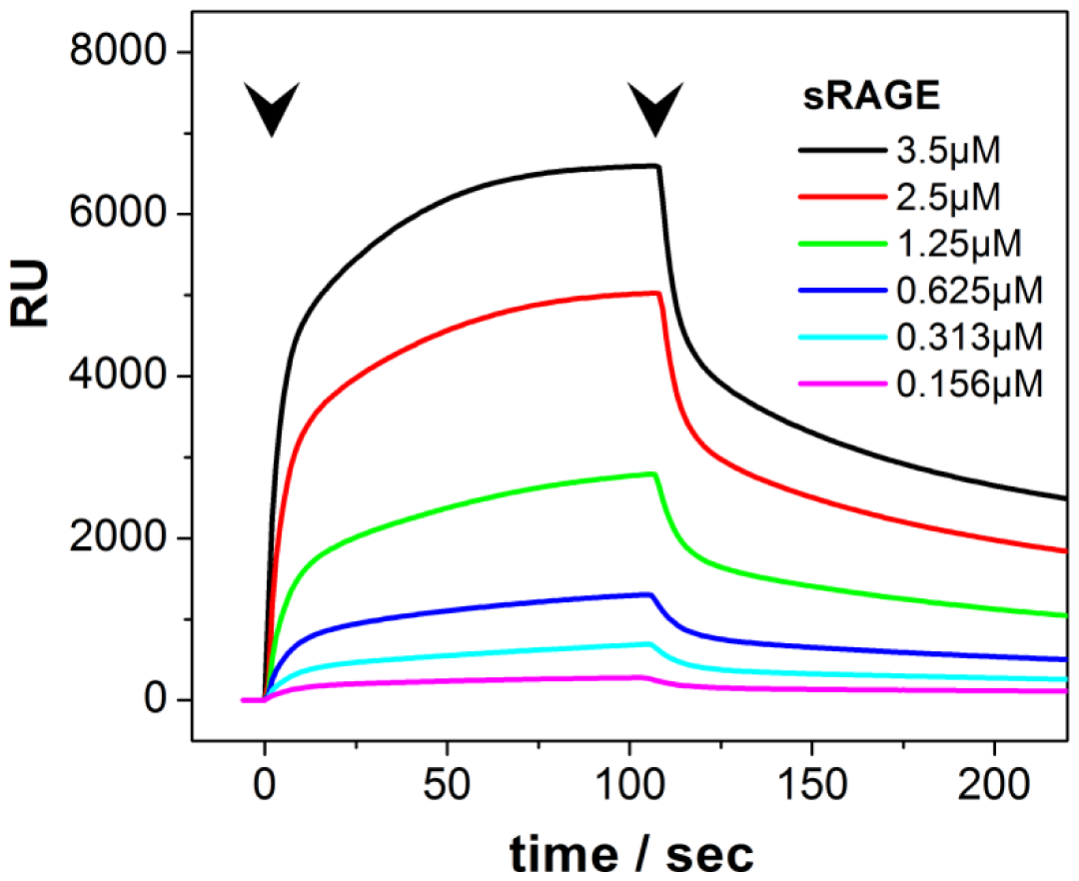
Direct RAGE-RAGE binding. Surface plasmon resonance analysis of homophilic interaction between soluble sRAGE and sRAGE immobilized on a Ni-NTA sensor chip. Soluble sRAGE was injected over the sensor chip at a flow rate of 10 µl/min. The arrows indicate the start and end of injections.

In order to further characterize RAGE homophilic interaction we performed surface plasmon resonance experiments. Direct RAGE-RAGE binding was observed using recombinant isolated proteins. The high affinity interaction of sRAGE of the mobile phase with sRAGE immobilized on the chip (Fig. 8) displayed a *K*_d_ = 0.47±0.07 µM. This value is in the range of affinities reported for homophilic interactions between other Ig-type cell adhesion molecules. Surface plasmon measurements revealed that CEACAM1 interaction is about 16-fold weaker with a *K*_d_ = 7.65 µM [52] whereas NCAM exhibits an almost 20-fold higher affinity with *K*_d_ = 0.025 µM [53].

### RAGE Mediates Homophilic Cell-cell Adhesion

**Figure 9:**
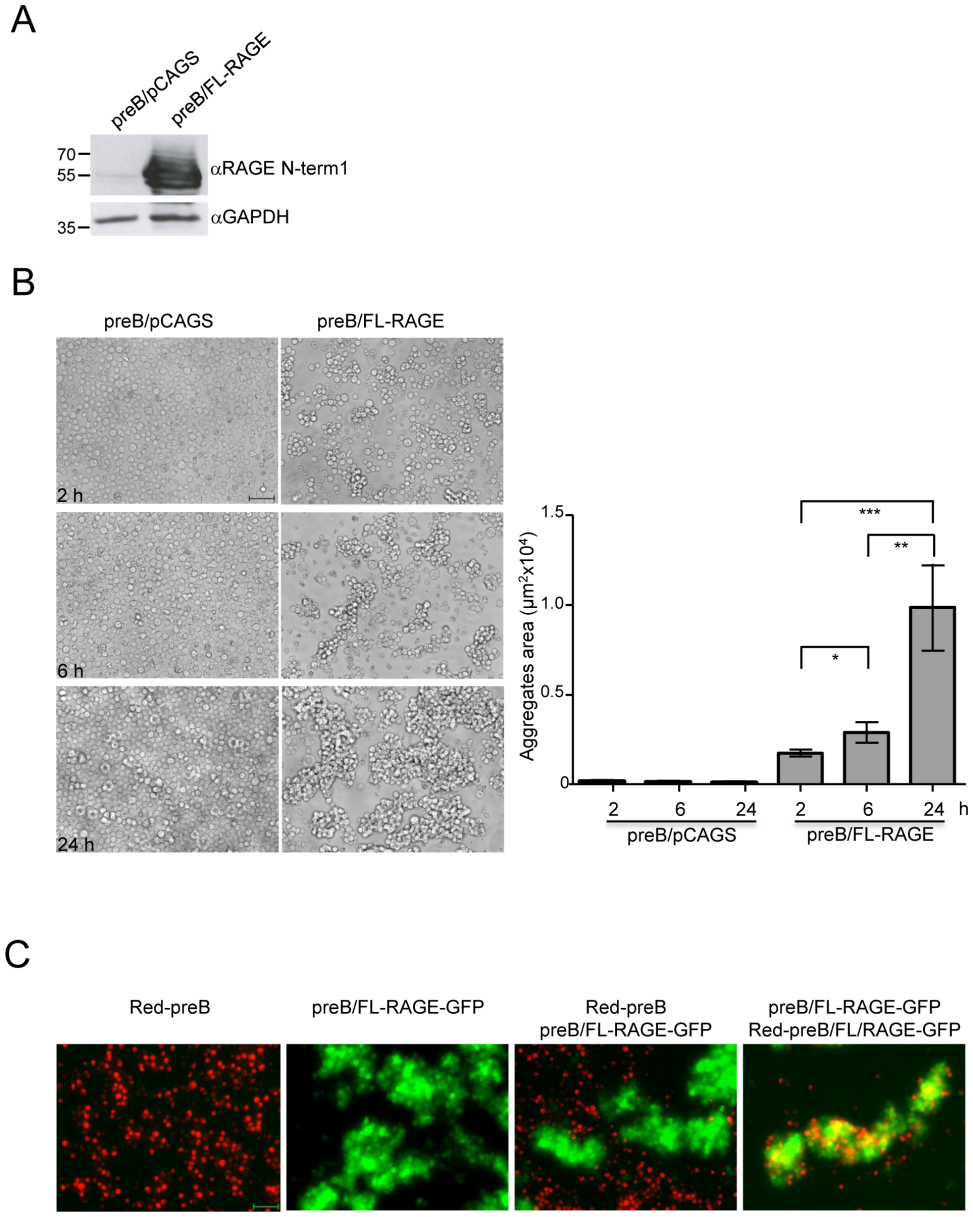
RAGE expression mediates cell aggregation through homophilic interactions. (**A**) Western blot analysis on control preB 300.19 cells (preB/pCAGS) or expressing FL-RAGE (preB/FL-RAGE) using αRAGE N-term1 antibody. Forty µg of cell lysate were loaded and detection of GAPDH was used as loading control**.** (**B**) Cell aggregation assay. preB/pCAGS or preB/FL-RAGE cells were cultured for 2, 6 or 24 hours before being photographed at 20× magnification in phase contrast (left panel). Bar corresponds to 50 µm. Quantification of cell aggregates area (right panel). Results are displayed as means±SEM (*, P<0.05; **, P<0.01; ***, P<0.0001). (**C**) Mixed cell aggregation assay. Red preB (Red-preB) or preB/FL-RAGE (expressing GFP; preB/FL-RAGE-GFP) cells were grown as single cell line suspension or mixed in equal number for 24 hours. Red-preB/FL-RAGE-GFP were mixed with preB/FL-RAGE-GFP for the same time. Bar corresponds to 50 µm.

To determine whether cellular expression of RAGE is able to promote homophilic interaction, we performed a cell aggregation for which cells growing in suspension are elective. We used the PreB 300.19 cell line transfected with an empty vector (preB/pCAGS) or stably expressing FL-RAGE (preB/FL-RAGE) (Fig. 9A). As shown in Fig. 9 B, during culturing preB/pCAGS grow as a suspensions of single cells, while preB/FL-RAGE cells clump together forming aggregates whose area increases in a time-dependent manner. To test whether the aggregation depends on homophilic interaction of RAGE molecules, we performed a “mixed” aggregation assay [37], [38] using green preB/FL-RAGE cells expressing GFP protein (preB/FL-RAGE-GFP), and preB 300.19 cells (that display same features of preB/pCAGS but do not express GFP) that fluoresce red. Thus, the two cell populations could be distinguished after mixing. As shown in Fig. 9C, preB 300.19 cells neither aggregate together nor with cells expressing RAGE, demonstrating that the presence of RAGE on two adjacent cells is necessary to adhere with one another. Indeed, when preB/FL-RAGE were labelled red (preB/FL-RAGE-GFP-red) and mixed with unlabelled preB/FL-RAGE-GFP, the two cell populations clumped together forming mixed aggregates (Fig. 9C). Altogether, these data indicate that membrane expression of RAGE contributes to cell-cell adhesion through homophilic interactions.

## Conclusion

Our analysis indicates that RAGE belongs to a group comprising ALCAM, BCAM and MCAM proteins, which are all cell adhesion molecules. In particular:

1. The *Ager*, *Alcam, Bcam, Mcam* genes share a similar genomic organization suggesting that they evolved from a common ancestor gene. *Ager*, the gene coding for RAGE, first appeared with Mammalia, and is part of a locus that originates earlier in evolution with Metazoans. In human, this locus belongs to the major histocompatibility complex (MHC) Class III region on chromosome 6p21.31 [54], which contains genes involved in the innate immune system, inflammation and regulation of immunity [55].
2. RAGE is very closely related to the adhesion proteins ALCAM, BCAM and MCAM as shown by structural alignments and further suggested by structural models and shares key structural features with these molecules.
3. RAGE shows properties expected of an adhesion molecule. In particular, cells expressing RAGE adhere to ECM components and to each other through homophilic interactions. On the other hand, mouse embryonic fibroblasts (MEFs) derived from *Rage−/−* mice exhibit reduced capability to adhere to ECM proteins compared to wild type cells (not shown). Structural models also suggest the RAGE-RAGE homophilic interactions *in trans* occur via the V-domain.

Notably, RAGE expression was previously shown to be induced on activated endothelial and epithelial cells, in particular after injury [56], [57], and to bind the CD11b/Mac-1 protein on leukocytes, aiding their extravasation [57], [58]. These activities suggest that RAGE can act as an Ig-cell adhesion molecule (Ig-CAM) [59] in the context of leukocyte extravasation. Furthermore, as it occurs for other CAMs [60], RAGE is cleaved by ADAM10 [35] and the shedding might influence its adhesive properties by regulating membrane expression. A common feature of many adhesion molecules is their tendency to form clusters to facilitate cell-cell contacts [61]. It has been proposed that RAGE assembles into constitutive oligomers within the plasma membrane [15] that after stabilization by ligands binding are able to activate RAGE-dependent signaling pathways [24], [62]. Thus, RAGE oligomerization *in cis* and *in trans* may play a crucial role in RAGE-mediated cell-cell or cell-matrix adhesion as well.

In conclusion, we suggest that RAGE was ancestrally a CAM, and only secondarily became a sensor involved in inflammation. Given its basal high expression in alveolar cells, RAGE might still serve as a cell adhesion protein in lung as well as in other cells/tissues when its expression is up-regulated in response to trauma-induced inflammation. This interpretation of RAGE’s functions might help in understanding its roles in pathologic conditions.
